# Investigating Community Pharmacist Experiences with Telepharmacy in the Absence of Regulatory Support in Indonesia

**DOI:** 10.1007/s44197-025-00368-z

**Published:** 2025-05-15

**Authors:** Imam Fathorrahman, Umi Athiyah, Abdul Rahem, Long Chiau Ming, Elil Renganathan, Yaser Mohammed Al-Worafi, Andi Hermansyah

**Affiliations:** 1 Doctoral study program, Faculty of Pharmacy, Universitas Airlangga, Surabaya, 60115 Indonesia; 2 Department of Pharmacy Practice, Faculty of Pharmacy, Universitas Airlangga, Surabaya, 60115 Indonesia; 3 Department of Medical Sciences, School of Medical and Life Sciences, Sunway University, Sunway City, 47500 Malaysia; 4 College of Medical Sciences, Azal University for Human Development, Sana’a, Yemen; 5 College of Pharmacy, University of Science and Technology of Fujairah, Fujairah, United Arab Emirates

**Keywords:** Telepharmacy, Pharmaceutical care, Community Pharmacy, Healthcare

## Abstract

**Background and Objectives:**

Telepharmacy has been increasingly used in Indonesian community pharmacies despite the absence of a policy regulating the services. In tandem with the lack of standardized pharmaceutical care, providing telepharmacy services may vary across community pharmacies. This study investigates the contemporary practice of telepharmacy in Indonesian community pharmacy.

**Methods:**

A cross-sectional survey using a validated online questionnaire was conducted. The targeted participants were community pharmacists who claimed to have provided telepharmacy service daily. The participants were approached using purposive sampling and extended using the accidental sampling method. The questionnaire asked about several activities that pharmacists do when delivering telepharmacy services. The data were subsequently analyzed using descriptive statistics.

**Results:**

Overall, 250 pharmacists participated in the online survey. Most respondents were female (73.6%) and less than 41 years old (78.6%). Despite respondents claiming to know telepharmacy (70%), more than half (52%) never attended any training and workshops on telepharmacy. Chat messaging apps were common platforms for telepharmacy (87.2%). Low patient uptake was evident in most pharmacies (74.4%). More than 96% of respondents ensured the accuracy of patient data before delivering the service. This includes verifying patient prescriptions and checking the prescribed medicines with patient history. However, fewer pharmacists frequently documented patient data (36%), communicated care plans to patients (22%), provided drug information (2.9%), and monitored outcomes (29.2%).

**Conclusion:**

The lack of regulation has contributed to unstandardized telepharmacy practice. Despite the untapped potential, the growth of telepharmacy services in Indonesian community pharmacies is uncertain, with ongoing support from the regulation needed.

**Supplementary Information:**

The online version contains supplementary material available at 10.1007/s44197-025-00368-z.

## Introduction

Indonesia’s healthcare system has faced significant challenges in providing equitable access to medical and pharmacy services, particularly in remote and underserved areas [1, 2]. It is, therefore, not surprising that telehealth and mobile health applications have become the alternative avenue for enhancing healthcare delivery [3]. The country has been familiar with mobile applications for managing pharmaceutical supplies and medical devices to ensure availability even in the most remote healthcare facilities [4, 5]. However, expanding access to pharmacy services through telepharmacy has been a new approach to pharmacy practice in Indonesia [6, 7].

The growing interest in telepharmacy, particularly after the COVID-19 outbreak, has led to the increasing use of the services in Indonesian community pharmacies [8–10]. However, the implementation of the service has demonstrated mixed responses. On the one hand, the adoption of telepharmacy and its digital features have been limited due to feasibility and patient issues. For instance, the usability of a telepharmacy app to help patients understand their medications has been very poor, reflecting a problem with adoption [11]. Likewise, the use of an integrated electronic healthcare service for rural areas has been hampered by the educational level of the patients [12]. On the other hand, telepharmacy has been shown to improve medication adherence in patients with chronic disease [13], help screen tuberculosis cases [14], and identify and manage chemotherapy-related symptoms in children with acute lymphoblastic leukemia (ALL) [15]. In addition, a study observing the perceptions of COVID-19 self-isolating patients revealed that telemedicine and telepharmacy have been pivotal for patient care during the pandemic [16].

There is no doubt that telepharmacy services in Indonesia can potentially address the healthcare delivery gap. Indeed, community pharmacists can play a more significant role in providing safe, cost-effective, and accessible telepharmacy care. Community pharmacists constituted the largest portion of the Indonesian pharmacist workforce [17], often becoming the first port of call for patient contact and the gatekeeper in primary care [18], not to mention the capacity for long-life learning and training, including for delivering telepharmacy care [19]. The problem, however, lies in the absence of policy support regulating the practice of telepharmacy in tandem with no standard available for delivering remote pharmaceutical care.

The lack of consistent and transparent legal regulations on implementing, applying, and supporting telepharmacy practice has been cited as the main barrier to telepharmacy care in most countries. For example, although telepharmacy is permitted in 28 states in the US, the varifying regulations between state jurisdictions have imposed burdensome restrictions on the practice [20]. In addition, the absence of a set standard has driven different telepharmacy models, which can hamper proper care delivery [21]. The situation can be more challenging in countries with no telepharmacy regulations. Studies from several countries reported that the implementation of telepharmacy has been slow, affecting its integration and adoption in community care [22–24], a situation similar to Indonesia.

Before the COVID-19 pandemic, telemedicine services had been implemented on a limited scale between selected healthcare facilities in Indonesia under the Ministry of Health (MoH) decree 20/2019 [22]. During the pandemic, the MoH allowed doctors and dentists to provide specific treatments, particularly online consultation and online medication review through telemedicine, to prevent the spread of the COVID-19 virus [23]. The introduction of the latest Health Law 17/2023 has shed light on providing telecare [24]. Whilst it has become the underpinning policy for telecare, no further subordinate legislation has been made despite the emerging practice across the country. Therefore, this study aimed to investigate the contemporary practice of telepharmacy in Indonesian community pharmacies by identifying the daily activities and practices of community pharmacists delivering telepharmacy care and exploring the potential future direction for telepharmacy regulation.

## Methods

### Study Design

A cross-sectional online survey was conducted among Indonesian community pharmacists between October 2023 and February 2024. Participants’ data were collected from the Indonesian Pharmacist Association network across 34 provinces in Indonesia.

### Study Population, Sample Size and Sampling Technique

At the time of the study, there were 32,091 community pharmacies nationwide [25]. Using the formula: n = [Z^2^P (1 − P)]/e^2^, where Z = value from standard normal distribution corresponding to desired confidence level (Z = 1.96 for 95% confidence level), e = 5% margin of error and *P* = 50% of proportion population, this study calculated a minimum of 380 respondents. The respondents were recruited using purposive sampling and extended using the accidental sampling method.

### Inclusion and Exclusion Criteria

The community pharmacist in this study was defined as working in the community pharmacy for a minimum of four days a week and involved in telepharmacy services. This study excluded individuals who declined to participate and participants who submitted incomplete and duplicated responses.

### Instrument Development

A questionnaire was developed as a data collection instrument. The questionnaire was created based on the literature review in the context of telepharmacy practice and regulatory setting, the standard of pharmacist care according to MoH decree 73/2016 [26], and the adoption of the pharmaceutical care concept as developed by Hepler and Strand [27]. Three Indonesian academics and practicing pharmacists attended a group discussion to assess the questionnaire for content validity. The questionnaire was then piloted to 30 pharmacists for face validity and reliability testing. Minor changes were made regarding the wording and question order. The pilot study resulted in a valid (*p* > 0.6) and reliable instrument (Cronbach α > 0.7) [28]. The final questionnaire consisted of two parts: the socio-demographic profile of the respondents and the characteristics of telepharmacy services. It included ten questions identifying the type and frequency of telepharmacy activities.

### Data Collection Procedure

Initially, the link to participate in the online survey was distributed to the representatives of pharmacist associations in 34 provinces in Indonesia. Several targeted pharmacy networks, including a group of community pharmacists and a group of pharmacists working in chain pharmacies, also received links for the survey. The link contains a questionnaire and a message explaining the research proposal, participant criteria, and consent for participation. Later, the link was posted on social media and shared with other pharmacist groups via WhatsApp©^,^ allowing for the accidental involvement of pharmacists in the survey.

### Statistical Analysis

A descriptive analysis was conducted with categorical variables presented as frequencies and percentages. Given the simple data management, statistical analysis was performed using Microsoft Excel© 2019.

### Ethical Considerations

The study has received ethics approval from the Human Research Ethics Committee of the Faculty of Pharmacy Universitas Airlangga No. 42/LE/2023. Participants provided informed consent before accessing the questionnaire.

## Results

Two hundred and fifty pharmacists completed the survey. The socio-demographic profile of the respondents is summarized in Table 1. The majority of respondents were female (73.6%), and three-quarters were aged less than 41 years old (76.8%) and had been working for more than 8 years as a pharmacist (34.4%). Most respondents worked in Java Island, with respondents from West Java, Central Java, and East Java constituting 56% of the sample. Most pharmacies only had one pharmacist (52%), and pharmacies relied upon private insurance (74.8%) as a means of customers paying for the pharmacy services (Table 1). Apart from the findings that the majority perceived adequate knowledge about telepharmacy (70%), more than half (52%) never attended any training or workshops on delivering telepharmacy services.

**Table 1:** Socio-demographic profile of the respondents (*n* = 250)

Characteristics	*n* (%)
Gender	
Male Female	66 (26.4) 184 (73.6)
Age (in years)	
20–30 31–40 41–50 51–60 > 60	88 (35.2) 104 (41.6) 46 (18.4) 11 (4.4) 1 (0.4)
Years of experience	
< 3 3–5 6–8 > 8	43 (17.2) 68 (27.2) 53 (21.2) 86 (34.4)
Type of pharmacy ownership	
Self-owned State-owned Investor-owned	82 (32.8) 35 (14) 133 (53.2)
Province	
Nangroe Aceh Darussalam West Sumatera South Sumatera Riau Islands Bangka Belitung Bengkulu Jambi Jakarta Capital City Banten West Java Central Java Yogyakarta East Java Bali West Nusa Tenggara East Nusa Tenggara Maluku West Kalimantan East Kalimantan South Kalimantan North Sulawesi South Sulawesi Papua West Papua	2 (0.8) 5 (2) 7 (2.8) 2 (0.8) 1 (0.4) 4 (1.6) 1 (0.4) 8 (3.2) 4 (1.6) 49 (19.6) 41 (16.4) 10 (4) 50 (20) 1 (0.4) 2 (0.8) 3 (1.2) 1 (0.4) 9 (3.6) 5 (2) 15 (6) 3 (1.2) 22 (8.8) 4 (1.6) 1 (0.4)
Number of pharmacists working in the pharmacy	
One More than one	130 (52) 120 (48)
Type of customer coverage	
National health insurance Private insurance Both	0 187 (74.8) 63 (25.2)
How knowledgeable are you about telepharmacy?	
Very knowledgeable Adequate knowledge Little knowledge to none	73 (29.2) 175 (70) 2 (0.8)
Have you ever attended training or workshop on telepharmacy?	
Yes No	118 (47.2) 132 (52.8)

Table 2 shows that chat messaging apps such as WhatsApp, Line, Telegram, and social media messenger have been the most common way to deliver telepharmacy services (87.2%). On the contrary, only 2% of the respondents used mobile apps for telepharmacy. Respondents claimed that drug information services and drug monitoring are the two most common services provided by telepharmacy, with 27.4% and 27.1%, respectively. Less than twenty patients have received telepharmacy care in most pharmacies (74.4%).

**Table 2:** Characteristics of telepharmacy services (*n* = 250)

Characteristic	*n* (%)
Type of platform for delivering telepharmacy	
Chat messaging Apps (WhatsApp, Line, and others) Telephone Others (mobile apps)	218 (87.2) 27 (10.8) 5 (2)
Frequently used telepharmacy services within the past year	
Dispensing medication Drug information service Patient counselling Patient assessment and need for drug therapy Medication adherence and adverse effect monitoring Homecare pharmacy remote follow-up	21 (8.5) 68 (27.4) 44 (17.7) 32 (12.7) 68 (27.1) 16 (6.6)
Number of patients receiving telepharmacy per month	
< 20 patients 20–50 patients > 50 patients	186 (74.4) 56 (22.4) 8 (3.2)

Table 3 highlighted ten activities comprising telepharmacy care. Respondents very frequently and frequently conducted three major activities, namely receiving the order and confirming patient data (96.4%), conducting legal checks and interpretation of the prescription (96.8%), and analyzing drug therapy problems (84.8%). In contrast, the pharmacists have limited or less frequently conducted the remaining seven activities. Interestingly, more than two-thirds (69.2%) of the respondents did not provide drug information, which fits the data in Table 2 regarding frequently used services. Conducting technical activities such as preparing e.g. packing medicine and checking the delivery progress was also not popular among pharmacists who deliver telepharmacy since 68% have never done it.

**Table 3:** Type and frequency of activities provided under telepharmacy services (*n* = 250)

Type of activities	Frequency of activities (*n*, %)			
	Very frequently	Frequently	Occasionally	Never
Receiving orders and confirming patient data	198 (79.2)	43 (17.2)	9 (3.6)	0
Checking prescriptions (including legal checks) and interpreting the patient’s condition	192 (76.8)	50 (20)	7 (2.8)	1 (0.4)
Analyzing drug therapy problems	118 (47.2)	94 (37.6)	34 (13.6)	4 (1.6)
Verifying drug therapy problems and consulting with other professionals	50 (20)	85 (34)	67 (26.8)	48 (19.2)
Documenting patient data	33 (13.2)	90 (36)	56 (22.3)	71 (28.4)
Communicating the care plan to the patient	15 (6)	55 (22)	98 (39.2)	82 (32.8)
Preparing e.g. packing medicine and checking the progress of delivery via courier service	9 (3.6)	20 (8)	52 (20.4)	170 (68)
Providing instructions and drug information	4 (1.6)	6 (2.4)	67 (26.8)	173 (69.2)
Monitoring and reporting outcomes	19 (7.6)	73 (29.2)	96 (38.4)	62 (24.8)
Following up and communicating the progress of the therapy to the patient	48 (19.2)	104 (41.6)	60 (24)	38 (15.2)

## Discussion

This study is among the first to explore community pharmacist experiences delivering telepharmacy services in Indonesia. Past studies have been focused on identifying pharmacist views and knowledge about telepharmacy [29], pharmacist willingness and readiness to provide telepharmacy [6, 7], or experimental approach conducted in a confined setting [13, 30], i.e., hospital excluding the role and responsibilities of community pharmacist which constituted the largest portion of pharmacist workforce in Indonesia.

The fact that most respondents who provided telepharmacy services in this study were early and mid-career pharmacists, as indicated by their age group, highlighted that telepharmacy is not a new concept to these groups. Telepharmacy relies on digital and remote technology; therefore, the younger pharmacist is better off delivering it. Younger pharmacists are technology adopters since they are more prepared to learn new skills for their career plans [31].This situation is not different from other countries as young pharmacists in developed and developing worlds confirm their willingness to embrace technological advancements on pharmacy confirm their willingness to embrace technological advancements in pharmacy [32–34]. Nevertheless, this finding underscores the consequence of rapidly changing technology realities and labor markets in pharmacy practice. Many young pharmacists must keep up with the rapid workforce and workplace changes. This has forced them to re-invent practice and how the work is carried out [35, 36].

Whilst this study found that respondents claimed to be knowledgeable about telepharmacy, they had never undertaken any formal training or workshops on telepharmacy. This finding speculated two key issues. Firstly, there is a possibility that the pharmacist in this study acquired practical skills in telepharmacy through self-taught. Pharmacists might have adjusted their conventional pharmaceutical care into a digital approach regardless of the quality and level of care they delivered. Technology’s pervasiveness and affordance may have enabled pharmacists to transform practice or, at the very least, simply topping up their services with a mere digital approach. Self-learning (also known as autodidactism or self-study) may come with risk, particularly in the case of the pharmacist who is responsible for direct patient care [37]. Pharmacists who self-learned about telepharmacy without proper guidance or role models may end up learning from weak or flawed references. The uncertainty in choosing the right learning material is the first challenge in self-learning. Pharmacists can also be overwhelmed by the overabundance of learning materials, particularly from online platforms, and find it challenging to learn them systematically and effectively. The problem of self-learning can be more significant when the variables of time constraints, self-motivation or interest, and lack of evaluation affect the learning process. In addition, self-learning might also facilitate variation in practice, given the lack of standards regulating telepharmacy practice. Secondly, the lack of training in telepharmacy in Indonesia was evident. Most pharmacist continuing education programs, workshops, and training focus on knowledge delivery and soft-skills development [38, 39]. Practical skills training in using telepharmacy has been scarce. In addition, the current curricula did not adequately prepare future pharmacists to embrace remote care, such as telepharmacy [29, 40]. This implies the need to provide training on telepharmacy and, more importantly, prepare students for a new digital care approach. Acquiring new skills in demand is critical for the survival of pharmacist work.

The respondents have used chat messaging apps to support telepharmacy services. This finding is similar to the practice of telepharmacy in most countries, including Iran [41], Canada [42] and Vietnam [43]. While using chat messenger can be effective for telepharmacy care, this may come with collateral consequences, particularly considering the low uptake of the service. For instance, pharmacist communication with patients might be limited during texting and chatting. A full body of information might get distorted and not to mention the lack of human interaction as compared to other platforms such as telephone or video conference. The poor communication might lead to low satisfaction and trust, poor understanding and failure of the therapy [44]. This will create backlash to the pharmacy with fewer people used the service. In addition, it is also worth mentioning that chat system can happen 24/7. This will not only present extra burden to the pharmacist workload but also may affect the image of the pharmacy if non-responsive to chat inquiries.

The respondents frequently delivered only three essential activities concerning telepharmacy service, leaving the remaining seven underutilized. On the one hand, this finding may highlight that pharmacists generally performed well in ensuring patient data accuracy, conducting prescription legal checks and interpretation, and analyzing drug therapy problems accordingly. Undoubtedly, these activities are determinants for setting further care plans for the patient. However, this may reflect that patients might not get the optimal care from contemporary telepharmacy services. For instance, respondents could not document and provide drug information to the patient. Apart from the fact that respondents mostly used chat messaging apps with which drug information delivery is perhaps limited, this finding suggests that documentation and communication with patients have been poor. Poor documentation and communication may lead to weak analysis and evaluation, eventually affecting the treatment outcome [45]. It can also retaliate to the aforementioned everyday activities since they also rely on proper documentation.

The limited provision of the other seven activities assumed that collaboration, documentation, and care communication, rooted in patient-centered care philosophy, were underdeveloped in the contemporary telepharmacy practice. The importance and relevance of patient-centered care have been broadly accepted in pharmacy [46]. This finding holds several implications for telepharmacy practice. Patients may receive different care preferences and expectations depending on their desired pharmacist interactions during each care encounter. Community pharmacy networks miss the optimal role and degree of connection with other health professionals in caring for the population. For the health system in general, the likelihood of getting positive clinical and societal outcomes from telepharmacy may be averted. As a result, the expectation to align seamless health care that improves quality, processes, and cost-effectiveness can be compromised. These implications have suggested the need for a standardized telepharmacy practice.

Several studies have reviewed the type of pharmacy services often delivered to patients under telepharmacy [44, 47–49]. Remote consultations, patient education, virtual care and monitoring, home delivery of medicine, and remote dispensing and supervision are the most common telepharmacy initiatives. Some of these services were already observed in this study, although no standard or guidelines are available in Indonesia. These services were designed to complement or as an adjunct to the traditional in-person pharmacy services. For instance, this study recorded that a small portion of respondents would follow up on the progress of homecare pharmacy visits. This finding reflects that telepharmacy is now perceived as an essential service to support the viability of pharmacy businesses, particularly given the absence of payment or reimbursement models. Community pharmacists should also determine patient preferences and eligibility before delivering telepharmacy. Patients who require intensive pharmaceutical care, have access to the internet and have sufficient technological literacy and communication skills would benefit most from telepharmacy services. Training and continuing education on telepharmacy might help pharmacists improve service performance and navigate them to find the most effective and efficient care model in a time of crisis compounded by the lack of regulations. However, in the long term, unless they were encouraged by legal regulations, this role development might not be sustainable [50].

Overall, this study suggested the need for a supportive regulatory landscape regarding telepharmacy. The Pharmacy Practice Act of 2009 and the Ministry of Health Directives 73/2016 regarding the standard of pharmacy services have been the underpinning policy regulating community pharmacy practice in Indonesia [17]. These legislations have been influential in regulating pharmacy services, predominantly focusing on managing pharmaceuticals and providing clinical pharmacy services [18]. Telepharmacy was adopted and implemented under these legislations and mainly delivered as an adjunct to conventional pharmaceutical care [51]. However, the emerging market for telepharmacy has implied the need for specific regulatory frameworks. The regulation may come in several forms; setting a standard of practice can be the stepping stone. In addition, introducing a model of care that fits the needs and demands of telepharmacy care would fill the gap in unstandardized telepharmacy delivery. It is fair to say that the existing governing policies and regulations do not adequately address the growing industry. The lack of legislation regulating the setup and operation of telepharmacy services at regional and national scales is a missed opportunity. The lack of uniform and comprehensive regulatory frameworks specific to telepharmacy may present risks in the safe handling of medicine and patient safety [44, 47]. This may exacerbate the contemporary practice in Indonesia, which is already daunted by complex and intractable issues of pharmaceutical care delivery.

Indonesia is amongst other developing countries which have introduced telepharmacy, such as Brazil [52], China [53], Iran [41], Jordan [54], Pakistan [55], Philippines [56], Sub-Saharan African Countries [57, 58] and Sri Lanka [59]. These countries have some innovative models of pharmacists delivering services from the use of social media apps to support medication consultation and clinical pharmacy interventions to reduce adverse drug events to practice collaboration with other health professionals. However, there does not appear to be a uniform approach towards legislation and regulation in telepharmacy. For example, Brazil and China have multiple legislative and regulatory references, which is the complete opposite of the absence of regulatory framework in the African and South-Asian countries [21, 47, 60]. This contrasting situation can be primarily attributed to the fact that telepharmacy is still developing in terms of practice and regulation in these countries [49]. Piecemeal policy approach or inceremental order often dominated the governance of regulations in these countries due to the nature of top-down policy development. For instance, Brazil and China have adopted and updated several guidelines and regulations over time [48]. However, the remaining countries including Indonesia are in pursuit of policy model that fit to their health system performance. In fact, most of these countries were even struggled to determine the e-health legislation which acts as the umbrella policy for telepharmacy. Such heterogeneity illustrates that telepharmacy regulation is still a proof of concept in major developing countries.

While this study has provided valuable insights into pharmacists’ experiences in delivering telepharmacy, the survey relied on self-reported data via the online platform. This might provide two main biases. Firstly, the self-report can introduce biases where participants overestimate their activities for telepharmacy. Secondly, online surveys would only reach participants who were more tech-savvy or comfortable with digital platforms. This method would exclude participants unfamiliar with online surveys or unable to access the surveys. In addition, this study primarily investigated the activities of selected pharmacists using a non-probability sampling technique. The participants may not fully mirror the national profile, potentially affecting the generalizability of the findings across the nation.

## Conclusion

The contemporary telepharmacy practice in Indonesia has been portrayed by the domination of early and mid-career pharmacists who have not participated in training or workshops on telepharmacy. The high reliance on chat messenger apps as the primary platform for service delivery, in tandem with low patient uptake and underutilized services, would imply the need for regulatory support. Telepharmacy offers untapped potential for broadening access to pharmacy services in Indonesia. However, a lack of regulation might affect the quality of care and thus jeopardize patient safety.

## Electronic Supplementary Material

Below is the link to the electronic supplementary material.

Supplementary Material 1

## Data Availability

No datasets were generated or analysed during the current study.

## Abbreviations

MoH: Ministry of Health
ALL: Acute Lymphoblastic Leukemia
