# Supplementary material for: Investigating Community Pharmacist Experiences with Telepharmacy in the Absence of Regulatory Support in Indonesia
Source: J Epidemiol Glob Health. 2025 May 15;15(1):71. doi: 10.1007/s44197-025-00368-z (PMC12081811; doi:10.1007/s44197-025-00368-z)
Supplement: Supplementary file 1 — Supplementary Material 1 [file 44197_2025_368_MOESM1_ESM.docx]

I understand that all information generated in this survey is anonymous and will be processed in aggregate/group data form so that it does not identify the name of a particular pharmacy or pharmacist. I also understand that the information generated in this survey is confidential and will be stored and used in accordance with ethical provisions. If you agree to participate in this survey, please provide a check mark **(√ )** in the column below

YesNo. You do not need to continue completing this survey.

**Please fill out the questions or choose one answer (√) that is appropriate.**

## RESPONDENT IDENTITY

| 1. | Name | : | apt ……………………………………….. |
| --- | --- | --- | --- |
| 2. | Gender | : |  Male  Female |
| 3. | Age | : | … year |
| 4. | Phone number | : | ………….............................................. |
| 5. | Email | : | …………………………..………...…. |
| 6. | Years of experience as a pharmacist in a community pharmacy | : |  < 3 year   3-5 year   6-8 year   > 8 year |
| 7. | Weekly attendance rate in the community pharmacy | : | < 2 times/week  2-3 times/week  4-5 times/week  >5 times/week |

- 1. **COMMUNITY PHARMACY IDENTITY**

| 1. | Pharmacy name |  | ………………………………………. |
| --- | --- | --- | --- |
| 2. | Address | : | Jl …………………………………….  ………………………………………. City/region: …………………… Province: …………………… |
| 3. | Ownership | : |  Self-owned   Investor-owned   State-owned |
| 4. | Number of pharmacists working in the community pharmacy | : | …… |
| 5. | Type of customer using telepharmacy service | : | National Health Insurance (BPJS)  Private insurance  Both |

### The following questions will determine your experience regarding telepharmacy services in community pharmacies.

1. How much do you know about telepharmacy services in community pharmacy?

Very knowledgeable

Adequate knowledge

Little knowledge

No knowledge

1. What media do you use to practice telepharmacy services in the pharmacy? (you may choose more than one answer)

Telephone or audio call platform

Video call platform

Chat-messaging platform

Mail-based software

For other platforms, please mention:

1. How many patients do you provide telepharmacy services to on a monthly average?

 < 20 patients

 20-50 patients

 > 50 patients

1. What types of telepharmacy services have you provided frequently within the past year?

 Dispensing medication

 Drug information service

 Patient counselling

 Others, please mention:

1. Have you ever attended training or workshop on telepharmacy?

Yes, please mention the name/title………………………….

Never

### The following question aims to determine the type and frequency of telepharmacy services.

Instruction

Give a check mark (√) on one of the options that corresponds to the telepharmacy practice you provided.

**VF = Very frequently**

**F = Frequently**

**O = Occasionally**

**N = Never**

| **No** | **Question** | **VF** | **F** | **O** | **N** |
| --- | --- | --- | --- | --- | --- |
| 1 | Do you ensure the accuracy of patient data when receiving orders / confirming patient data? |  |  |  |  |
| 2 | Do you check and ensure that the prescriptions (including legal checks) are appropriate to the patient’s condition / interpret ingthe patient’s condition |  |  |  |  |
| 3 | Do you identify and analyze drug therapy problems? |  |  |  |  |
| 4 | Do you verify and consult drug therapy problems with other professionals? |  |  |  |  |
| 5 | Do you document patient data? |  |  |  |  |
| 6 | Do you communicate the care plan to the patient? |  |  |  |  |
| 7 | Do you prepare medicine and check the progress of delivery via courier service? |  |  |  |  |
| 8 | Do you provide instructions and drug information? |  |  |  |  |
| 9 | Do you monitor and report patient outcomes? |  |  |  |  |
| 10 | Do you follow up and communicate the progress of the therapy to the patient? |  |  |  |  |
